# Dose- and Time-Dependent Effects of a *Ferula communis* Extract on Bone Remodelling in a Human Osteosarcoma Cell Line

**DOI:** 10.3390/molecules31203597

**Published:** 2026-10-10

**Authors:** Jessica Maiuolo, Miriam Scicchitano, Lorenza Guarnieri, Stefano Ruga, Francesca Bosco, Francesca Oppedisano, Rosamaria Caminiti, Valeria Mazza, Saverio Nucera, Ernesto Palma, Carolina Muscoli, Vincenzo Mollace

**Affiliations:** 1 Department of Health Science, Institute of Research for Food Safety & Health (IRC-FSH), University “Magna Graecia” of Catanzaro, 88100 Catanzaro, Italy; maiuolo@unicz.it (J.M.); r.caminiti@unicz.it (R.C.); valeria.mazza@unicz.it (V.M.); saverio.nucera@hotmail.it (S.N.); palma@unicz.it (E.P.); muscoli@unicz.it (C.M.); mollace@libero.it (V.M.); 2 Department of Health Science, University “Magna Graecia” of Catanzaro, 88100 Catanzaro, Italy; lorenza.guarnieri@unicz.it (L.G.); rugast1@gmail.com (S.R.); francesca.bosco@unicz.it (F.B.)

**Keywords:** *Ferula communis*, Saos-2 cells, osteoporosis, RANKL/OPG proteins, bone remodelling, osteoblasts/osteoclasts, conditioned medium

## Abstract

*Ferula communis* is a herbaceous plant in the family Apiaceae and order Apiales. It has two chemotypes that cannot be distinguished botanically, although one is highly toxic while the other is not. The non-poisonous chemotype has many beneficial activities for human health, including antioxidant, anti-inflammatory, cytotoxic, antimycobacterial, antiproliferative, neuroprotective, and estrogenic properties, thanks to its numerous secondary metabolites. This manuscript evaluates the effects of a *Ferula communis* extract on bone remodelling in a human osteosarcoma cell line. The extract from *Ferula communis* (FER-E) comes from the roots of the plant and, after chemically removing toxic ferulenol, further chromatographic analysis allowed us to estimate the ferutinin content, titrated at 25%. Saos-2 cells were treated with FER-E for both short times (24, 48, and 72 h) and longer times (up to 20 days) and, at each time, three aspects were evaluated: (a) cell viability; (b) cytosolic calcium concentration; and (c) the expression of specific proteins (OPG-RANKL) related to bone remodelling. The results were encouraging, suggesting that FER-E effects were dose- and time-dependent. Furthermore, both short and prolonged treatments positively modulated bone structure maintenance. For example, treatment with FER-E for 72 h preserves osteoblastic activity, presumably at the expense of osteoclastic activity. Treatment for 12 days seems to turn off this activity. Still, surprisingly, in a time window between the thirteenth and twentieth day, the cells produce one or more late compounds, capable of returning to the extract the ability to activate osteoblastic activity again. If these findings are properly confirmed, both in vitro and in vivo, *Ferula communis* could be associated with the delicate mechanism of bone remodelling.

## 1. Introduction

*Ferula communis* is a perennial herbaceous plant, a dicotyledonous angiosperm, belonging to the family *Apiaceae* and the order Apiales. This plant is present throughout the Mediterranean area and extends east to the Arabian Peninsula and south to Tanzania [1,2]. Precisely because of the very hot climate in which it grows, *Ferula communis* is a “summer resting plant”, which takes a vital break in the warmer months, interrupting growth, losing its leaves, and drying out the above-ground part. This apparent condition, similar to death, is a defensive mechanism of the plant that protects the roots and prevents it from expending energy to flower again in spring [3]. When *Ferula communis* is cut, broken, or chewed, each portion produces sulfur-based compounds that release an unpleasant odour; this evolutionary strategy helps the plant defend itself from herbivores: in fact, the sulfur-based compounds negatively alter its flavour and discourage predators. A botanical classification of *Ferula communis* is represented in Figure 1.

### 1.1. Botanical Description

Botanically, the root of *Ferula communis* is a woody taproot that descends very deep into the soil. It functions as a reservoir of water and nutrients that nourish the plant, allowing the above-ground part to dry out in summer and be reborn in spring. The stem is erect, cylindrical, robust and light; it is used by craftsmen to build objects such as chairs, baskets, cages and stools [4]. The leaves of *Ferula communis* change according to their position on the plant. The basal leaves are the largest (up to 100 cm) to collect sunlight. At the base, leaves have a wide sheath that envelops the stem [5]. The flowers of *Ferula communis* are small, yellow, and gathered in large umbrella-shaped inflorescences. At the base of the petals of each flower is a small structure called a “nectar disc” that produces and accumulates nectar. This disc is very shiny and visible, and these properties attract pollinating insects and some migratory birds that feed on the sugary liquid directly from the flowers [6]. The fruit of *Ferula communis* is an obovoid-compressed diachene about 1.5 cm long. It is very light and is easily dispersed by the wind [7]. A botanical description of *Ferula communis* is represented in Figure 2.

### 1.2. Toxicity of Ferula communis

Some plants can be toxic because they produce secondary metabolites, such as alkaloids, terpenes, glycosides, and oxalates, that can be harmful to those who consume them [8,9]. Therefore, these plants can be considered antinutritional and responsible for a wide range of deleterious effects [10]; they must be excluded from animal feeding to avoid accidental poisoning [11,12]. It is believed that there are two chemotypes of *Ferula communis*, which cannot be distinguished botanically, although one is highly toxic while the other is not [13]. The toxic variety is considered responsible for a lethal hemorrhagic syndrome, also called “ferulosis”, caused by ferulenol, a prenylated coumarin [14]. The mechanism of action of ferulenol consists of acting as a competitive agent and a vitamin K antagonist. This activity prevents the production of prothrombin and other coagulation factors, inhibiting blood clotting [15]. Clinical signs appear within 24–48 h after consumption of the plant, and the most recurrent are increased clotting time, muscle weakness, haemorrhages in the mucous membranes, bleeding from the nostrils, and blood in the faeces, until the animal’s death [16,17].

### 1.3. Phytoestrogenic Properties of Ferula communis

The non-toxic chemotype is composed of natural sesquiterpenes, accumulated mainly in the roots and seeds of the plant. These secondary metabolites act together with other phytochemicals, causing cytotoxic or contrasting effects on tumour cell lines [18,19]. This chemotype includes compounds that possess interesting properties and perform beneficial activities for human health, including antioxidant, anti-inflammatory, cytotoxic, anti-mycobacterial, antiproliferative, neuroprotective and estrogenic properties [20,21,22]. Among these activities, estrogenic properties have generated interest, demonstrating a significant impact on biological systems related to the hormonal axis [23]. The molecule responsible for this estrogenic property is ferutinin, the main bioactive compound isolated from different parts of the *Ferula communis* plant, and it is an ester of a sesquiterpene alcohol with a daucanic-type structure [24]. Like most terpenes, ferutinin also demonstrates dose-dependent activity: low concentrations result in protective effects, while higher doses determine cytotoxic properties and selective mitochondrial dysfunction on tumour cell lines [25]. Due to its morphological and spatial similarity to steroid hormones and the presence of the phenolic group, ferutinin can mimic the estradiol molecule, showing a strong binding affinity for human estrogen receptors alpha and beta [26,27]. The phytoestrogenic activity of ferutinin can be appreciated in all estrogen-dependent tissues, and therefore also in bone remodelling. Throughout a woman’s fertile life, characterised by the constant presence of estrogens, bone renewal is finely regulated by the destruction of old tissue (through the action of osteoclasts) and the formation of new bone (carried out by osteoblasts). In particular, estrogens slow osteoclasts, turning off the signals that activate them, and protect osteoblasts, amplifying signals related to their survival. Additionally, estrogen helps the body retain calcium ions and vitamin D [28]. Biochemically, bone tissue balance depends on three key elements produced or managed by osteoblasts: (a) osteoprotegerin (OPG) is a protein that protects bones by blocking the action of osteoclasts, preventing calcium loss and subsequent fragility of bones, and maintaining skeletal balance and strength [29]. (b) RANK (Receptor Activator of Nuclear Factor κB) is a key membrane receptor expressed on precursors and mature osteoclasts. Osteoblasts indirectly regulate osteoclastogenesis by expressing RANKL on their membrane. (c) RANKL (RANK Ligand) is the protein that binds to the RANK receptor, stimulating its maturation and activation. When RANKL binds to the RANK receptor on osteoclast precursors, cellular signals for osteoclast differentiation, fusion, and survival are activated, stimulating bone breakdown [30]. To close the loop in regulating bone remodelling, OPG can bind to RANKL, instead of RANK, acting as a “bait” receptor, blocking the binding between RANKL and RANK, preventing osteoclast maturation and activation, and ultimately reducing bone resorption. Therefore, the OPG/RANK/RANKL triad is a system that strictly regulates bone remodelling, and its functioning is ensured by the production of estrogen hormones. When less estrogen is produced, or none at all, bone remodelling stops, and the body destroys more bone than it creates, leading to a loss of bone strength and onset of osteoporosis [31]. Hormone replacement therapy (HRT) is widely used to control and replace a sudden lack of estrogen. Since HRT can promote the onset of side effects, it would be better to use alternative estrogenic therapies with the same efficacy but with reduced side effects [32,33]. Numerous in vivo studies have demonstrated that ferutinin prevents osteoporosis by a dose-dependent mechanism, exerting osteoprotective effects, as evidenced by specific changes in bone remodelling in ovariectomized rats. Ferutinin is also known in biology and pharmacology for its strong activity as a calcium ionophore. This molecule can induce the release of calcium from the endoplasmic reticulum and its subsequent accumulation in mitochondria [34], thereby increasing the permeability of cellular and mitochondrial membranes to this ion. Increased calcium concentration, among other functions, intervenes in bone remodelling, as already mentioned. In this study, an extract of *Ferula communis* will be used, in which ferutinin is titrated to 25%. Since a plant extract comprises many chemical molecules that often act synergistically, enhancing the effect of a single molecule, improving total absorption and reducing undesirable effects [35], we hypothesise that *Ferula communis* extract may perform better than the single component ferutinin.

In light of the above, this manuscript aims to evaluate the effects of a *Ferula communis* extract obtained from the root of the plant in an in vitro model of human osteosarcoma cells (Saos-2). These cells exhibit properties similar to those of osteoblasts, representing an optimal model for the study of bone biology. In particular, we will evaluate the concentration- and time-dependent effects of the extract on cell viability, calcium ion modulation, and bone remodelling.

## 2. Results and Discussion

### 2.1. Administration of Short-Term FER-E and Effects on Saos-2 Cell Viability

Saos-2 cells were exposed to FER-E at different concentrations (0–2500 µg/mL) for 24, 48 and 72 h; the respective results are shown in Figure 3. As can be appreciated at each time point considered, the effects of FER-E demonstrate dose-dependent activity; in fact, low concentrations (5–40 µg/mL) do not produce deleterious effects, while higher doses (80–2500 µg/mL) have been associated with cytotoxic effects. Presumably, this result is conditioned by the presence of the sesquiterpene ferutinin, which, like most terpenes, causes concentration-dependent effects [25]. However, the various compounds of the extract can enhance and amplify the effects of ferutinin. The observed increase in toxicity was not attributable to the presence of the prenylated coumarin ferulenol, since this component was completely removed from the extract, as described and demonstrated in [36]. The effects of mortality caused by FER-E appear to increase significantly and gradually at treatment times 24, 48 and 72 h (Figure 3a–c); for example, the concentration 320 µg/mL determines a mortality of 28% after 24 h, 38% after 48 h and 65% after 72 h of treatment. In contrast, the results reported in Figure 3d are superimposable with those in Figure 3c, demonstrating that, after 72 h of treatment with FER-E, mortality is stabilised and has reached a plateau level.

### 2.2. Bone Remodelling Induced by FER-E Treatment

To study the estrogen-like effect of FER-E, we evaluated the expression of OPG and RANKL proteins, which are involved in bone remodelling, and the results are shown in Figure 4. It is important to highlight that a modulation of the expression of these proteins is not appreciable before three days of treatment. In fact, after 24 and 48 h, the modulation is still not complete and reproducible. Presumably, cells need the right time to get used to FER-E treatment and to perform protein transcription and translation [37]. In these experiments, we considered only low, non-toxic concentrations of FER-E. In fact, the higher concentrations induce toxicity, and the possible modulation of the RANKL-OPG binomial would not have achieved a phytotherapeutic purpose. The expression trend of the proteins of interest is the opposite: RANKL decreases as the FER-E concentration increases, demonstrating that there is a lower quantity of ligand capable of promoting osteoclastic destruction of the bone. Band quantification suggests a 10% reduction, from FER-E 10 µg/mL to FER-E 20 µg/mL, and a 12% reduction from FER-E 20 µg/mL to FER-E 40 µg/mL. Conversely, under the same experimental conditions, OPG increases its expression, highlighting a greater possibility of binding to RANKL and preventing bone loss [38,39]. In this case, band quantification suggests a 28% increase, from FER-E 10 µg/mL to FER-E 20 µg/mL, and a further 12% increase from FER-E 20 µg/mL to FER-E 40 µg/mL. These encouraging results suggest that FER-E at low concentrations stimulates osteoblasts, emphasising bone maintenance and its stability.

To date, it is known that the RANKL-OGP axis in bone remodelling is particularly influenced by oxidative stress, which is significantly elevated in menopause [40,41]. Polyphenols, with their known antioxidant properties, can help maintain osteoblast stability and support bone strength [42,43]. The strong antioxidant activity of FER-E has already been evaluated in a previous manuscript by our research group [2].

### 2.3. Calcium Ionophoretic Effect of FER-E

Treatment with FER-E increases the permeability of cell membranes to calcium ions, increasing their concentration in the cell. This characteristic is specific to some variants of *Ferula communis* [44,45], and the results are shown in Figure 5. Figure 5a shows cytosolic calcium curves following treatment with low, non-toxic concentrations of FER-E, compared to untreated cells. The most interesting portion of the curve is the linear trend preceding the sharp increase, indicating that cellular cytosolic calcium does not originate from the extracellular environment or mitochondria, as indicated in the corresponding section of “Materials and Methods” [46]. This consideration confirms that calcium derives from the reserves contained in the endoplasmic reticulum and that the reason capable of justifying its variations is attributable to FER-E treatment. The subsequent portions of the curve add nothing to the knowledge of cytosolic calcium and represent only evidence that the chosen measurement method is working.

Quantification of basal calcium concentration, reported in Figure 5a, is represented in Figure 5b and shows a significant increase proportional to the increase in FER-E dose. This increase is extremely important for osteoblasts, which use intracellular calcium as a key second messenger, regulating proliferation [47], differentiation [48], response to hormonal stimuli [49], and bone matrix synthesis [50]. More specifically, calcium carries cellular signals, regulates gene expression, stimulates communication between cells, ensures hormonal control, and controls mineral balance [51]. During menopause, the drop or absence of estrogen also reduces calcium absorption and accelerates bone loss, increasing the risk of osteoporosis [52]. Therefore, FER-E, through its estrogen-like property and through the ionophoretic effect of calcium, manages to safeguard bone health [53]. However, excess calcium in the cytoplasm of osteoblasts causes irreparable damage: (a) enters the mitochondria, destroying membrane potential and suppressing ATP production; (b) activates destructive enzymes, such as proteases and phospholipases; (c) increases the production of free radicals that damage biological macromolecules; (d) triggers apoptosis, compromising the formation of new bone tissue and promoting the risk of developing osteoporosis [54,55]. The results obtained highlighted that, as Fer-E concentrations increase, there is a gradual alteration with a reduction in cytosolic calcium coming from the endoplasmic reticulum. This effect is represented in Figure 5c,e, while its quantification is reported in Figure 5d. This result presumably indicates the consumption of calcium stores within this organelle, suggesting altered calcium permeability of mitochondrial membranes and subsequent toxicity, as commonly reported in the scientific literature [56]. Indeed, our research group has already tested the toxic effects of high doses of FER-E in an in vitro model of MCF-7 breast cancer cells, observing increased expression of the mitochondrial proteins Cytochrome C and Bax, both precursors of apoptotic death [2].

### 2.4. Chronic Effects of FER-E on Saos-2 Cell Viability

After demonstrating the dose-dependent effect of FER-E, we also investigated whether a time-dependent effect was active. So, we also decided to study any effects of FER-E administered for longer periods. Cells were maintained in culture as described, and treatments were performed every three days, as described in Section 3 (e.g., days 4, 7, 10, 13…). A representative diagram of the treatments with FER-E, in acute and chronic, is shown in Figure 6.

The results obtained were reported in Figure 7. As can be seen, the curves still show a double trend: the low concentrations of FER-E (2.5–40 µg/mL) are non-harmful and reproducible, while the high ones (80–2500 µg/mL) demonstrate significantly increasing toxicity. Untreated cells were also considered at all times and showed high reproducibility, indicating no variation. Expression of RANKL and OGP proteins was assessed at 12 days of FER-E treatment. As can be observed in Figure 7c, the modulation of these proteins is no longer visible at low concentrations of the extract during this period. Presumably, therefore, protection of bone remodelling is only guaranteed with acute treatment of FER-E. Figure 7d,e show the quantification of the RANKL and OPG bands, without significant variations, respectively.

### 2.5. Chronic Effects of FER-E on Cytosolic Calcium Concentration

Since we assessed cell viability and bone remodelling beyond three days of FER-E treatment, we did the same with cytosolic calcium modulation, as highlighted in Figure 8. The cytosolic calcium concentration, measured in untreated cells at 3, 6 and 12 days, was found to be equal, as demonstrated in Figure 8a, in which three superimposable curves can be visualised; furthermore, this trend can also be found in Figure 8b, reporting the same basal cytosolic calcium concentration. Cytosolic calcium measurements were also conducted at 12 days after FER-E treatment, and the results indicated a completely different modulation (Figure 8c). In fact, while the curves of untreated cells and those treated with the last non-harmful concentration are superimposable, the curves for high doses of FER-E show a significant reduction in basal calcium concentration. In fact, the last measurable concentration was 320 µg/mL, and the highest concentrations did not detect cellular cytosolic calcium. Figure 8d shows the respective quantification. Cytosolic calcium levels were also measured at 6 and 9 days; the results showed levels gradually higher than at 12 days, but the same trend.

However, it should be remembered that the calcium measured in this experimental model comes only from the endoplasmic reticulum. Under physiological conditions, there is a “cross-talk” between the endoplasmic reticulum and the mitochondria, and these organelles exchange a continuous, bidirectional flow of molecules and signals that regulate cellular homeostasis [57,58]. If calcium in the endoplasmic reticulum is reduced, the influx of calcium into mitochondria is also decreased; the main consequences are an energy deficit, metabolic alterations, and stress that can compromise the cell’s survival or specific functions [59,60]. Significantly increased mortality could easily be justified as a consequence of this ion reduction. Furthermore, the reduction in calcium, together with the reduced expression of proteins RANKL and OPG, disfavors bone remodelling.

### 2.6. What Happens on the Following Days of Treatment

After treatment with FER-E for 12 days, we would have expected the same trend at higher times, but surprisingly, with the treatment performed later, the effect of the extract changed significantly, as shown in Figure 9. The variation concerns the extent of toxicity at high concentrations (Figure 9a): in fact, we can observe significantly lower values compared to the toxicity achieved at 12 days of exposure to FER-E (Figure 9b). It is important to underline that the results obtained always show high toxicity, such as still identifying *Ferula communis* as a harmful plant, but it is reduced compared to shorter times. This phenomenon cannot be highlighted as appearing on a specific day, but rather from a certain period of time onwards: in general, we can state from 13 to 20 days, as can be appreciated from the high standard deviation in these experiments. It’s as if the treatment carried out between the thirteenth and twentieth day triggers something new that has never happened before. As can be appreciated, the increase in FER-E concentration corresponds to a significantly greater reduction in toxicity: for example, the mortality of FER-E 80 µg/mL at 12 days is reduced by 48% in the period between 13 and 20 days, while that of FER-E 2500 µg/mL is reduced, in the same period of time, by 70%.

### 2.7. Use of Conditioned Medium

Finding a single explanation that justifies this variation in toxicity is difficult; it is reasonable to hypothesise the following motivations:

(a) Cells must reach adequate levels of some specific factors (e.g., pH, metabolic capacity, cellular health status) [61,62,63,64].
(b) Cells need a certain time to metabolise harmful FER-E treatment and express a more tolerant response to toxicity [65].
(c) Cells produce some metabolites late to reduce the toxicity of FER-E [66].

Likely, all options considered are partially involved, but the possibility that a late metabolite could reduce FER-E-induced toxicity is extremely fascinating. Since we do not know the exact day when cells treated with FER-E can produce the “compound x” capable of reducing toxicity, we conducted a guiding experiment in which, after treatment with the extract, mortality was measured every day. In this experiment, mortality reduction occurred on day 17 of treatment, and we retained the respective growth medium to use it as a “conditioned medium”. The conditioned medium is a culture medium previously used for cell growth that has been enriched with growth factors, cytokines, exosomes, antioxidant metabolites, and signal molecules released from living cells [67,68]. A short time of exposure to a conditioned medium (for example, 6 h) could initiate some intracellular signalling pathways, but the synthesis and accumulation of protective proteins or the modulation of receptors could only be partial. For this reason, we chose longer exposure times (24 h) to allow the factors secreted in the medium to stably bind receptors, activate gene transcription, and accumulate antioxidant enzymes or cytoprotective molecules in quantities sufficient to counteract the toxic insult [69]. Experiments with the conditioned medium were conducted, and the results are represented in Figure 10. In Figure 10a, the cell viability curves are visible; in particular, it is possible to appreciate the curve of the treatment with FER-E at 12 days, colored in black, while the dotted black line is the curve corresponding to the same treatment (FER-E at 12 days) but also exposed for 24 h to the conditioned medium (with FER-E at 17 days). It’s as if toxic doses of FER-E caused significant stress to the cells, but the conditioned medium contained something capable of diluting the damage and building a mechanism to escape the unfortunate fate. For example, considering that numerous growth factors carry out intracellular survival signals, or anti-inflammatory cytokines that reduce oxidative stress and inflammation related to cellular damage, we can hypothesise that in this situation something similar occurs [70,71,72].

Similarly, cytosolic calcium levels tell us the same message: Figure 10b shows the cytosolic calcium levels of untreated cells at 12 days (in black) and cells treated with FER-E 320 µg/mL for 12 days (in red). The first profile is normal and indicates the good health of the cells; the second is extremely altered and indicates a very low level of cytosolic calcium, as well as a condition of cellular stress and suffering. Surprisingly, cytosolic calcium levels of cells treated with FER-E 320 µg/mL for 12 days and exposed for 24 h to conditioned medium (dashed red line, with FER-E at 17 days) have statistically increased.

In Figure 10c, the expression of RANKL and OGP proteins is shown after treatment with FER-E for 12 days and subsequent exposure to the conditioned medium for 24 h. This result is truly surprising and shows a modulation of protein expression that is superimposable on that obtained after three days of treatment with the extract. In this regard, it is possible to compare Figure 10c with Figure 4a. Furthermore, exposure to the conditioned medium was able to reactivate the expression of the same proteins, which were quenched following FER-E treatment for 12 days. In this regard, it is possible to compare Figure 7c with Figure 10c. Bone-like remodelling, promoted by FER-E, could therefore still occur after chronic treatment with the extract [73,74,75]. Only the concomitant presence of an osteoclastic cell line could confirm or reject this hypothesis [76,77,78].

It is difficult to justify the “miraculous effects” of the conditioned medium; based on scientific knowledge, we can only hypothesise what happens in our experimental model, and two events should be considered: (1) the type of damage induced; a cell population can survive and recover after prolonged toxic treatment only if the damage has not been irreversible. In fact, if DNA or cellular organelle damage is too severe, cells activate the programmed cell death program. Furthermore, in a cell population, some cells may enter a state of quiescence or temporary tolerance to drugs [79]. Cells can also develop resistance to toxic treatment through molecular adaptations that allow a cell population to survive a hostile environment. Cells can increase active efflux, thanks to membrane proteins that pump the toxic substance out of the cell before it causes damage. Finally, cells can also produce a greater quantity of enzymes capable of neutralising toxic molecules or quickly repairing the damage caused by treatment [80,81]. (2) At the same time, it is essential to use a high-quality conditioned medium that defends cells from toxic treatments thanks to its “secretome” rich in paracrine factors, which protect damaged cells from prolonged toxicity, activate molecular survival and repair pathways, inhibit apoptosis by stimulation of anti-apoptotic proteins, and block effector caspases that induce cell death. Growth factors in the conditioned medium may bind surface receptors of damaged cells, activating intracellular rescue pathways. Finally, many nutrients and signal molecules push the cell to restore the integrity of its membrane.

## 3. Materials and Methods

### 3.1. Material and Extraction Procedure

The FER-E extract was obtained from the root of the *Ferula communis* plant, collected in April 2022, in Macomer, a small town in the province of Nuoro (Sardinia, Italy), and properly preserved. The extraction protocol has been appropriately described and reported in [2]. The compound ferulenol was removed from FER-E to reduce the risk of related toxicity, as demonstrated by the HPLC spectra shown in [36]. A chromatographic analysis allowed us to estimate that ferutinin was contained in FER-E at 25%.

### 3.2. Cell Cultures and Treatments

Saos-2 cells, an osteosarcoma cell line, were purchased from the American Type Culture Collection (20099 Sesto San Giovanni, Milan, Italy). These cells were maintained in culture in Dulbecco’s modified Eagle’s medium (DMEM), enriched with 100 U/mL penicillin, 100 µg/mL streptomycin, 10% heat-inactivated fetal bovine serum (FBS), in a humidified 5% CO_2_ atmosphere at 37 °C. When the cells reached 50% confluence, they were treated with FER-E (5–2500 µg/mL). In experiments in which FER-E treatment was short-term, cells were incubated for 24, 48, or 72 h; in contrast, during longer treatment times, FER-E was added, freshly dissolved, every three days, as represented in Figure 6.

#### The Guiding Experiment

To confirm the hypothesis that the cells produced, after a certain period of treatment with the extract, one or more compounds responsible for the observed reduction in toxicity, we carried out the treatment with FER-E as described, and from the twelfth to the twentieth day, we measured the cell mortality daily. Since, in the guiding experiment, this phenomenon occurred on the seventeenth day, we stored and used this medium, considering it a conditioned medium. Subsequently, it was found that the production of these “protective compounds” occurred from the thirteenth to the twentieth day of treatment, depending on the cellular health.

### 3.3. Cell Viability

Cell viability was assessed by light microscopy using the trypan blue (0.4% *w*/*v*) exclusion assay [82]. Cells were seeded in 6-well plates at a density of 75 × 10^3^ cells/well. After treatment as described, cells were harvested, stained with Trypan blue, and observed under a microscope. The number of cells was assessed by direct counting with a hemocytometer. Cell mortality was reported as the percentage of stained (non-viable) cells compared to the total cells counted (composed of the sum of non-viable stained cells and viable non-stained cells).

### 3.4. Intracellular Calcium Measurements

Measurement of cellular calcium levels was performed using the rhodamine probe (Rhod 2, Molecular Probes, Fisher Scientific Italia, Milan, Italy) [83], which binds to the ion present in the cell and, when subjected to excitation at λ = 552 nm, emits a broad fluorescence. Saos-2 cells, at the end of treatment, were exposed to Rhod 2 for 1 h at 25 °C and protected from light. Subsequently, the cells were washed in a calcium- and magnesium-free solution to prevent calcium from entering the cells. The samples were subjected to flow cytometric analysis. After three readings—which provided the basal concentration of calcium ions—the cells were exposed to thapsigargin (1 μM for 200 s), a non-competitive inhibitor of a class of enzymes known by the acronym SERCA, responsible for increasing the cytosolic concentration of calcium from the endoplasmic reticulum. Under these conditions, three new flow cytometric readings were taken and, after 100 s, ethylene glycol-bis(β-aminoethyl ether)-N, N, N0N-tetraacetic acid (EGTA) was added at 500 μM to chelate calcium. It is worth noting that, before the readings, the cells were also treated with the uncoupler of mitochondrial oxidative phosphorylation, carbonyl-4- (trifluoromethoxy) phenylhydrazone (FCCP, 1 μM), as well as with the mitochondrial ATPase inhibitor, oligomycin (1 μg/mL). Both substances excluded the involvement of mitochondrial calcium. All flow cytometric detections were performed using a FACS Accuri apparatus (Becton Dickinson, Milan, Italy).

### 3.5. Cell Lysis and Immunoblot Analysis

Following treatment, cells were washed with cold PBS and lysed with a prewarmed buffer (at 80 °C) containing 50 mM Tris-HCl (pH 6.8), 2% SDS, and a mixture of protease inhibitors. Lysates were immediately inserted into water at 100 °C for 2 min, and protein concentration was determined by the DCA assay. After adding 0.05% blue bromophenol, 10% glycerol, and 2% β-mercaptoethanol, the samples were boiled again and loaded into a 15% polyacrylamide/SDS gel. Following electrophoresis, the polypeptides were transferred to nitrocellulose, and the filters were blocked with TTBS/milk (TBS 1%, Tween 20, and non-fat dry milk 5%). Antibodies were then used to reveal the respective antigens; the primary ones were incubated overnight at 4 °C, while the secondary antibodies, conjugated with horseradish peroxidase, were incubated for 1 h at room temperature. Blots were developed by chemiluminescence. The following primary antibodies were used: Rabbit Polyclonal RANKL antibody, (ab9957, Abcam, Prodotti Gianni–20138 Milan, Italy-dilution 1:1000); Mouse Monoclonal OPG Antibody (TR1BK1: sc-53976, Santa Cruz Biotechnology-00012 Guidonia Montecelio (RM), Italy-dilution 1:1500); Mouse monoclonal, AC-15 beta-actin Antibody (A5441 Sigma Aldrich, 20149 Milan, Italy-diluti’on 1:5000). Secondary antibodies were used at a dilution of 1:20,000.

## 4. Conclusions and Future Perspectives

The results obtained in this study are still preliminary and suggest that it would be interesting to delve deeper into this topic. In fact, the hypothesis that the plant can belatedly produce a compound/s capable of reducing the toxicity is very fascinating. Anyway, it is worth recognising the limitations of this research: the experimental model used, the Saos-2 cell line, represents a good in vitro model for studying bone biology, osteoblastic differentiation and extracellular matrix mineralisation. However, its tumour nature is far from the desired goal; for example, this osteosarcoma possesses significant genetic alterations [84], and its proliferation and survival pathways are altered compared to healthy osteoblasts [85]. Furthermore, bone remodelling is a highly dynamic process that requires a perfect balance between new bone formation (mediated by osteoblasts) and bone resorption (mediated by osteoclasts). This study, therefore, would also require the osteoclastic cell line [86].

So, the continuation of this manuscript provides:

1. To use a non-tumour osteoblast line;
2. To use a co-culture system, consisting of healthy osteoblasts and osteoclasts;
3. To identify the late component/s capable of reducing toxicity at prolonged treatment times. This further investigation could be carried out through metabolomics experiments on a suitable conditioned medium [82,83].
4. To extend this trial to in vivo models, to experience the effects of the extract of *Ferula communis* following the crossing of the gastrointestinal system. This step could provide the correct information for subsequent human assessment.

## Figures and Tables

**Figure 1:**
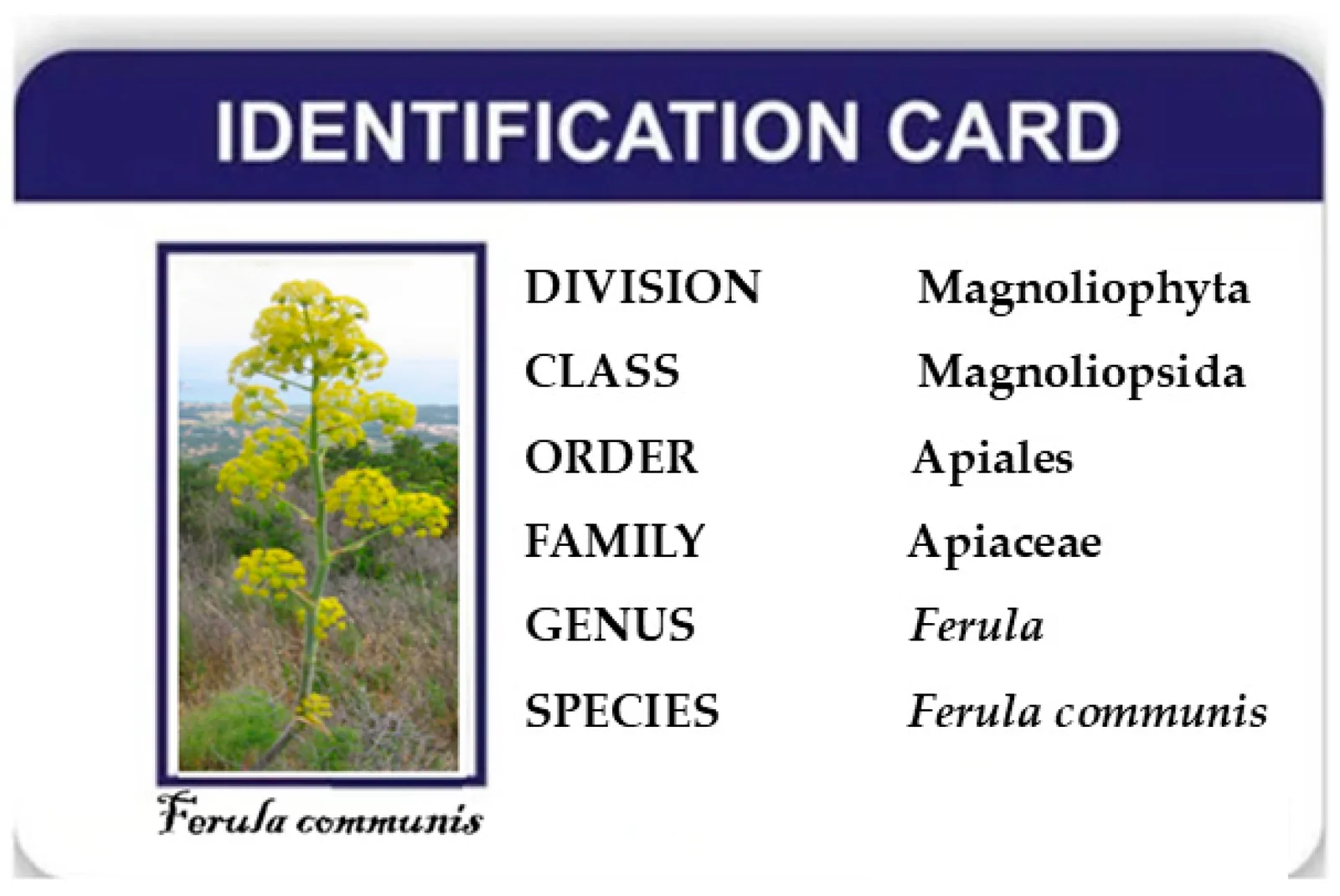
Botanical classification of *Ferula communis*.

**Figure 2:**
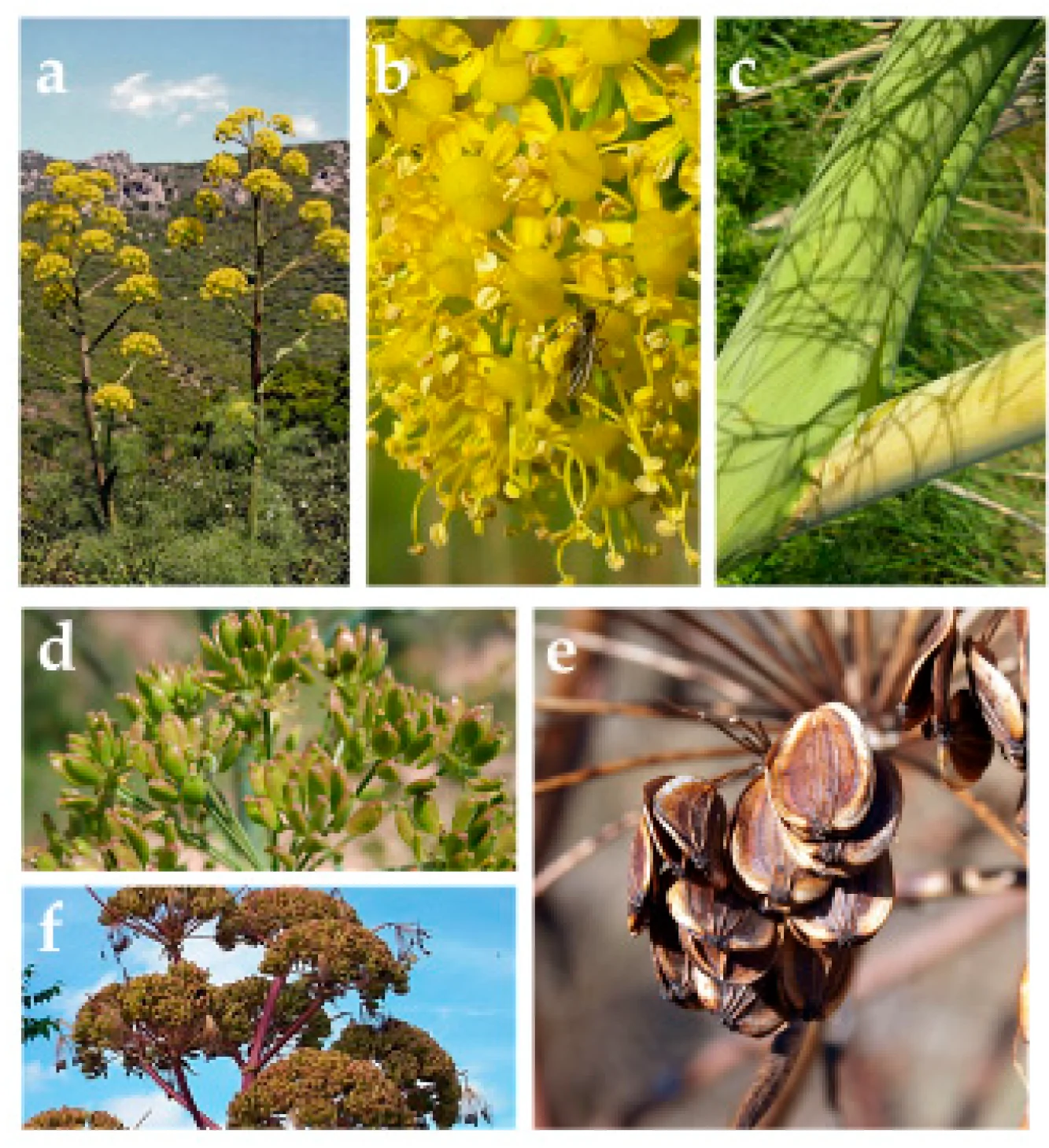
Botanical description of *Ferula communis.* (**a**) Plant with typical “umbrella” inflorescence; (**b**) Flowers with highlighted nectar disc; (**c**) Stem wrapped in a leaf sheath; (**d**) Fruits; (**e**) Brown dried fruits; (**f**) Dry inflorescence. This image was taken and edited from https://www.actaplantarum.org/flora/flora_info.php?id=12388 (accessed on 1 September 2026).

**Figure 3:**
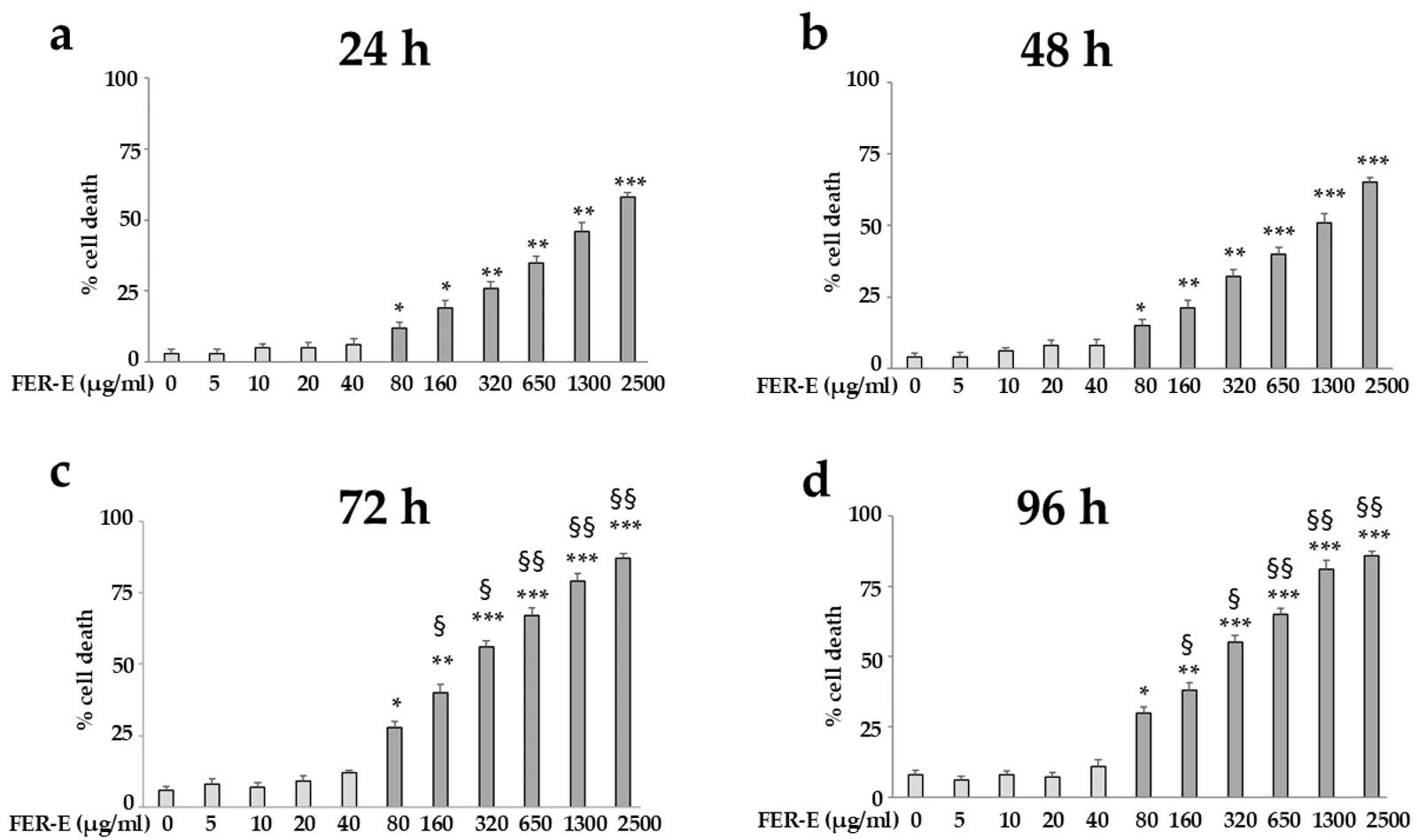
Effects of FER-E on Saos-2 cell viability. Cell mortality following treatment with FER-E for 24, 48, 72 and 96 h is represented in panels (**a**–**d**), respectively. In each panel, low concentrations are represented by light grey histograms; the high concentrations by dark grey histograms. Six independent experiments were performed, and the values are expressed as the mean ± SD. * denotes *p* < 0.05 vs. untreated cells; ** denotes *p* < 0.01 vs. untreated cells; *** denotes *p* < 0.001 vs. untreated cells; § denotes *p* < 0.05 vs. the same concentration after 24 h of treatment; §§ denotes *p* < 0.01 vs. the same concentration after 24 h of treatment. A Tukey–Kramer comparison test followed the analysis of variance (ANOVA).

**Figure 4:**
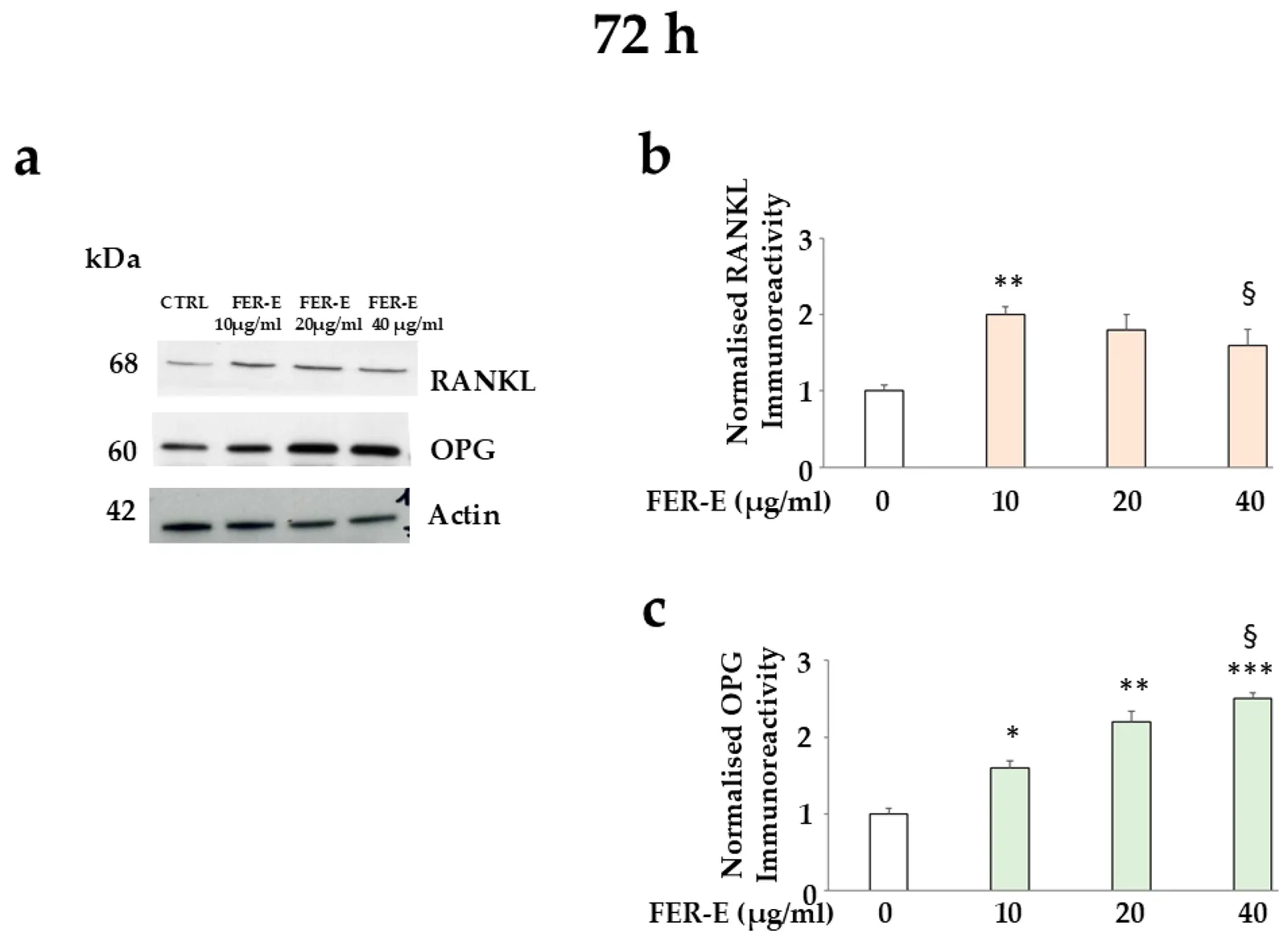
Expression of RANKL and OPG proteins following FER-E treatment. Modulation of the expression of the indicated proteins is represented in panel (**a**). The correctness of protein loading was validated by the protein Actin. Panels (**b**,**c**) show respective quantifications. Three independent experiments were performed, and the values are expressed as the mean ± SD. * denotes *p* < 0.05 vs. untreated cells; ** denotes *p* < 0.01 vs. untreated cells; *** denotes *p* < 0.001 vs. untreated cells; § denotes *p* < 0.05 vs. the concentration of FER-E 10 µg/mL. A Tukey–Kramer comparison test followed the analysis of variance (ANOVA).

**Figure 5:**
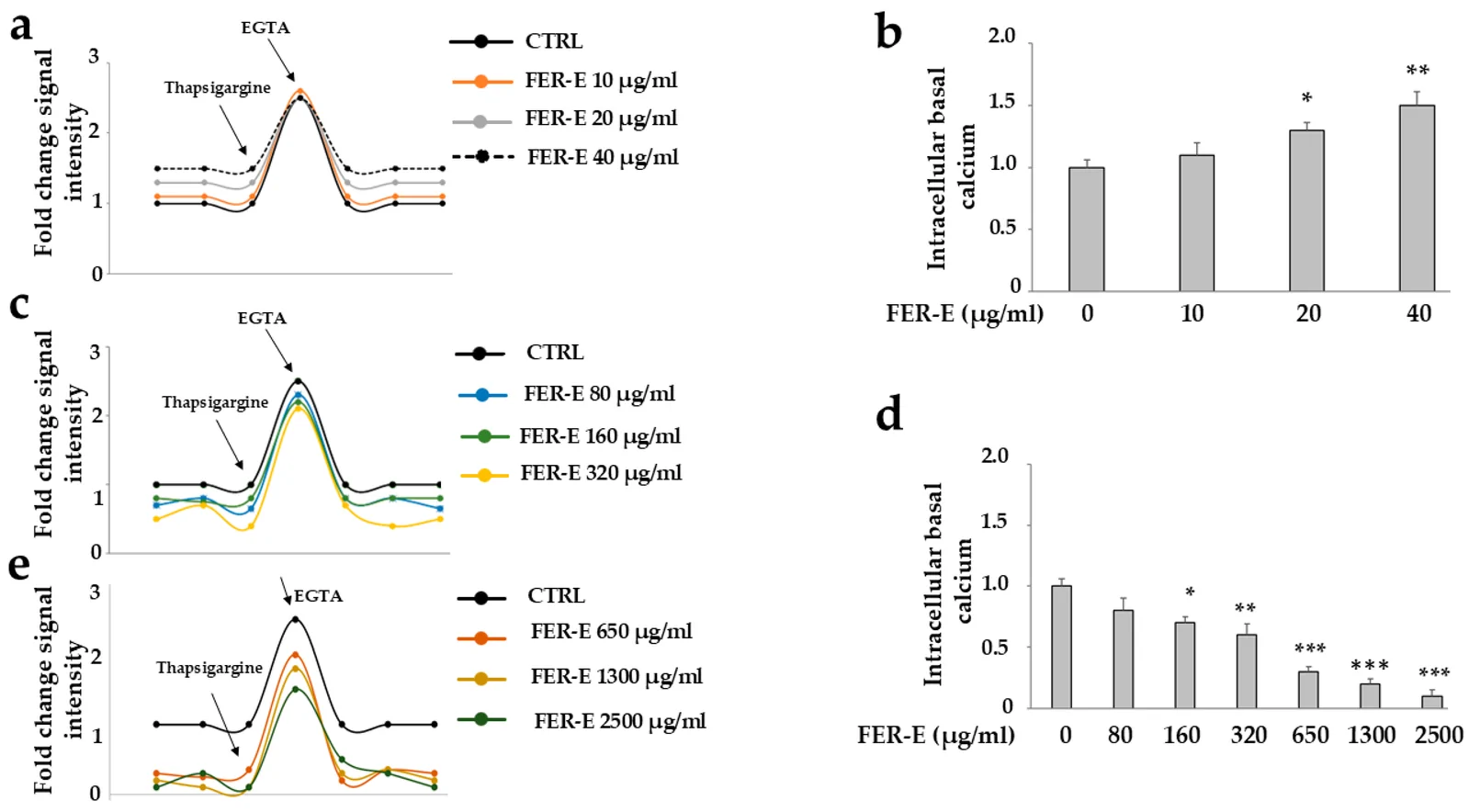
Quantification of cytosolic calcium after treatment with different concentrations of FER-E. In panels (**a**,**c**,**e**), the curves relating to cytosolic calcium levels are shown after treatment with FER-E. Panels (**b**,**d**) show the respective quantification. The arrow indicating treatment with thapsigargin, an ATPase-dependent calcium agonist, indicates the treatment of cells with this drug to induce an increase in cytosolic calcium from the endoplasmic reticulum. The arrow indicating treatment with ethylene glycol-bis (2-aminoethyl ether)-N, N, N′, N′-tetraacetic acid (EGTA), a calcium ion-selective chelating agent, indicates the treatment of cells with this drug to induce a reduction in cytosolic calcium. The use of thapsigargin and EGTA is essential to demonstrate that the chosen method for quantifying cytosolic calcium is working. Three independent experiments were performed, and values are expressed as mean ± SD. * indicates *p* < 0.05 compared to untreated cells; ** indicates *p* < 0.01 compared to untreated cells; *** indicates *p* < 0.001 compared to untreated cells. A Tukey–Kramer comparison test followed analysis of variance (ANOVA).

**Figure 6:**
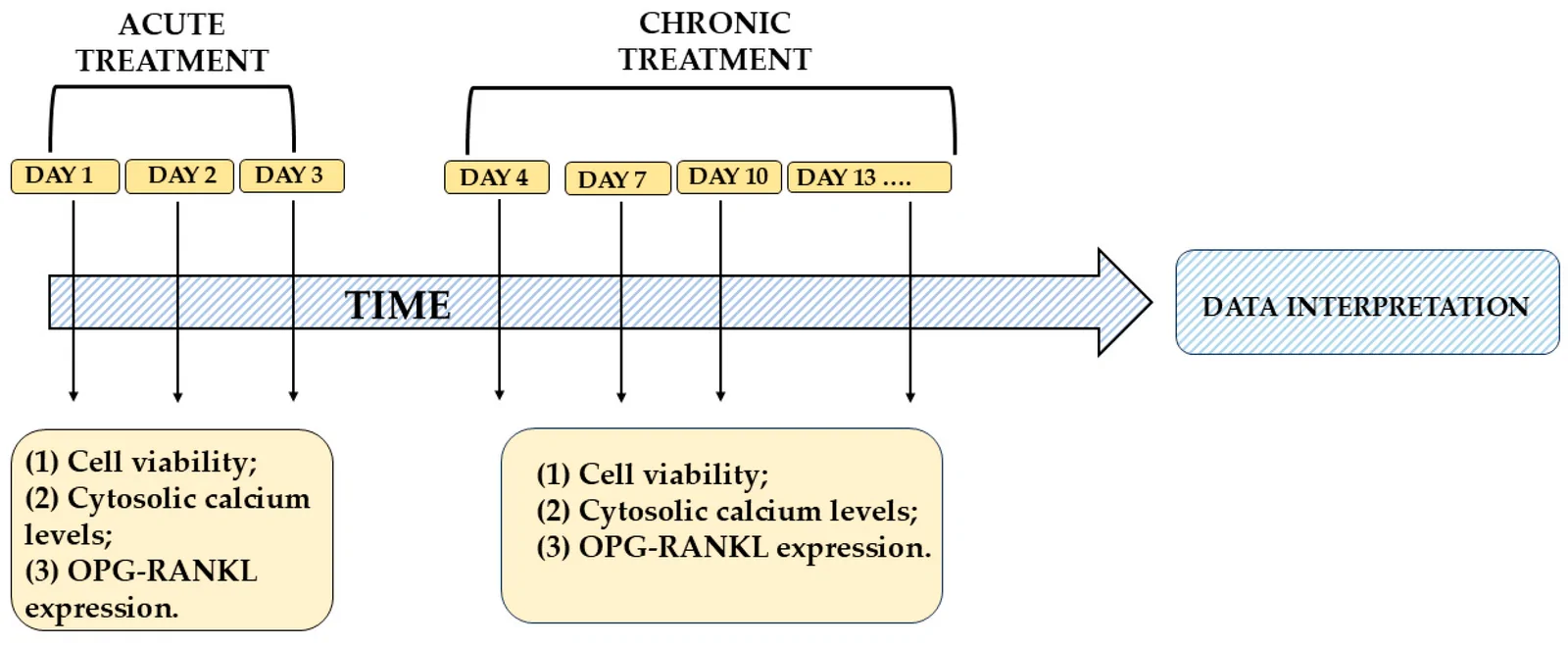
A representative diagram of the acute and chronic treatments with FER-E.

**Figure 7:**
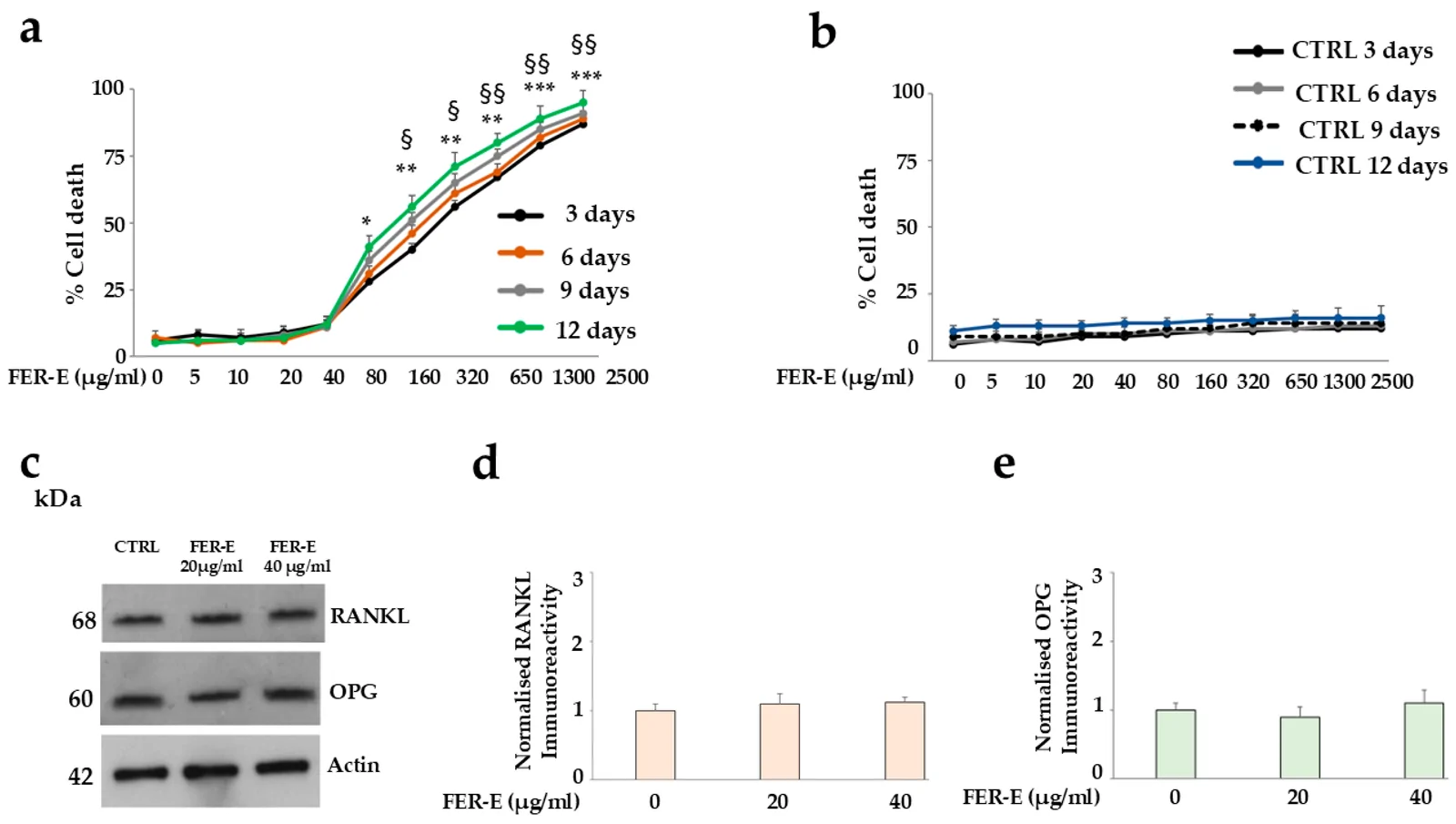
Chronic effects of FER-E on Saos-2 cell viability and bone remodelling. The results of treating the Saos-2 cell line with FER-E for an extended period were obtained. In particular, cells were treated with different concentrations of FER-E every three days. Panel (**a**) shows the curves obtained after 3, 6, 9, and 12 days. As can be appreciated, the curves show once again the dose-dependence already visualised at shorter times. In panel (**b**), untreated cells exposed only to the vehicle are represented. Panel (**c**) reports the expression of RANKL and OPG proteins at twelve days of FER-E treatment, obtained by Western blotting experiments. Finally, in panels (**d**,**e**), the respective quantifications are reported. Three independent experiments were performed, and the values are expressed as the mean ± SD. * denotes *p* < 0.05 vs. untreated cells; ** denotes *p* < 0.01 vs. untreated cells; *** denotes *p* < 0.001 vs. untreated cells. § denotes *p* < 0.05 vs. the first high concentration (80 µg/mL); §§ denotes *p* < 0.01 vs. the first high concentration (80 µg/mL). A Tukey–Kramer comparison test followed the analysis of variance (ANOVA).

**Figure 8:**
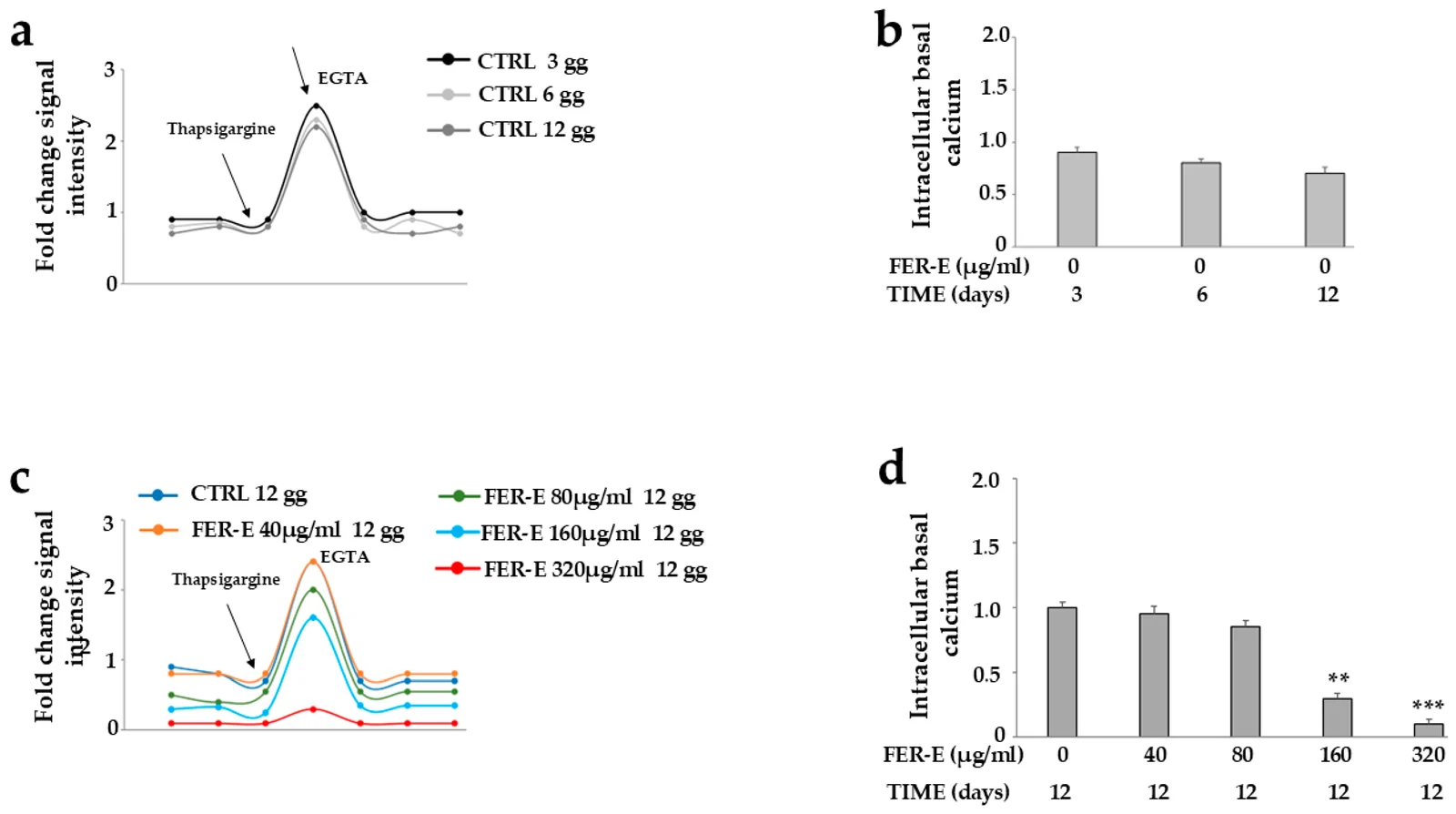
Chronic effects of FER-E on cytosolic calcium concentration. Panel (**a**) shows cytosolic calcium levels in untreated cells at 3, 6, and 12 days; panel (**b**) highlights the respective quantification. Panel (**c**) demonstrates the modulation of cytosolic calcium in cells treated for 12 days with increasing concentrations of FER-E. Finally, panel (**d**) shows the quantification of the results reported in panel (**c**). Three independent experiments were performed, and the values are expressed as the mean ± SD. ** denotes *p* < 0.01 vs. untreated cells; *** denotes *p* < 0.001 vs. untreated cells. A Tukey–Kramer comparison test followed the analysis of variance (ANOVA).

**Figure 9:**
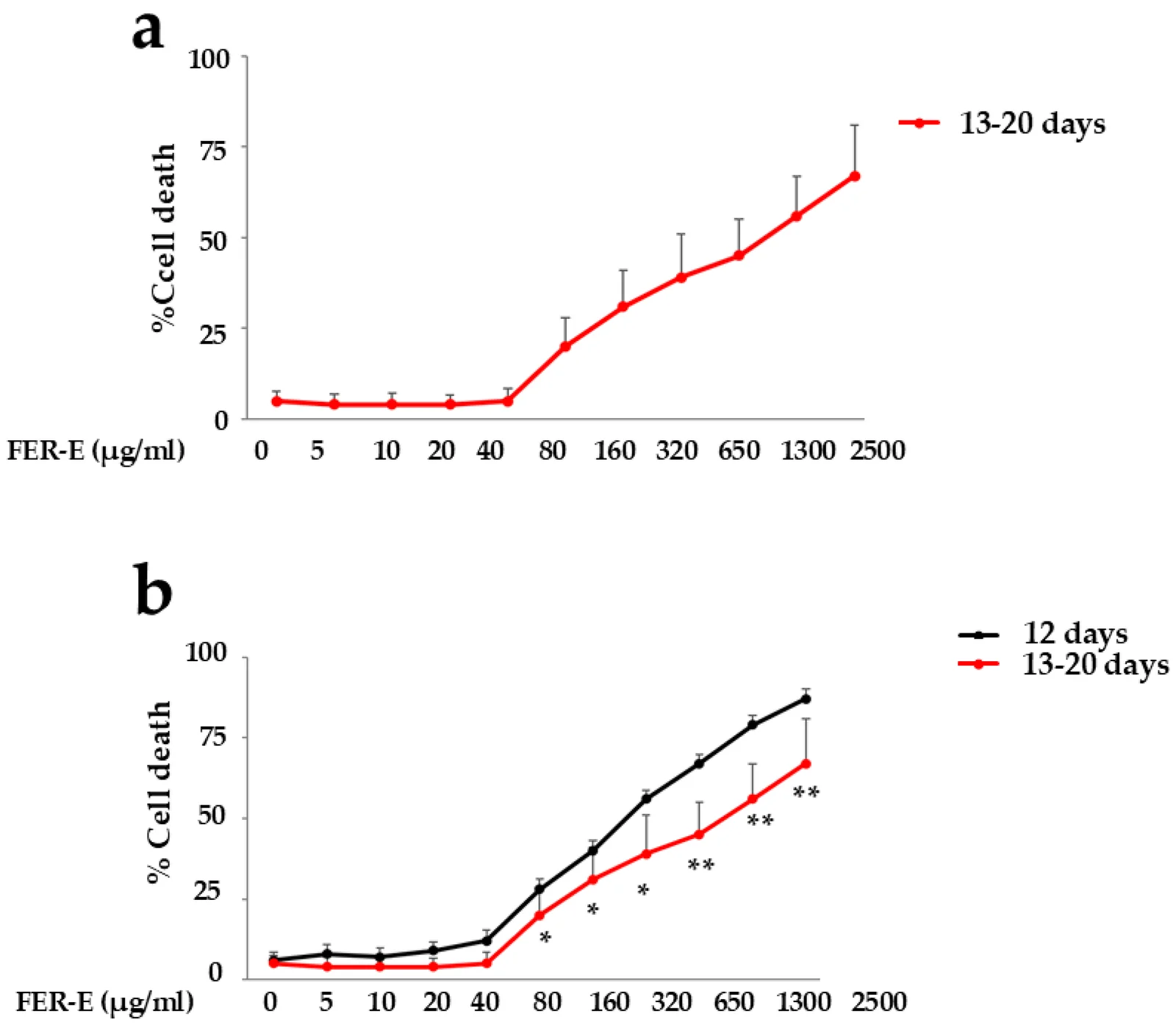
The toxicity of high FER-E concentrations is reduced in prolonged treatment times. Panel (**a**) shows the toxicity following FER-E treatment at 13–20 days, while panel (**b**) compares these values with those observed after FER-E treatment for 12 days. Many experiments had to be performed because this change appeared randomly between the thirteenth and twentieth day of FER-E treatment, increasing the standard deviation of the results. Twenty independent experiments were performed, and the values are expressed as the mean ± SD. * denotes *p* < 0.05 vs. the same concentration of FER-E at 12 days of treatment; ** denotes *p* < 0.01 vs. the same concentration of FER-E at 12 days of treatment. A Tukey–Kramer comparison test followed the analysis of variance (ANOVA).

**Figure 10:**
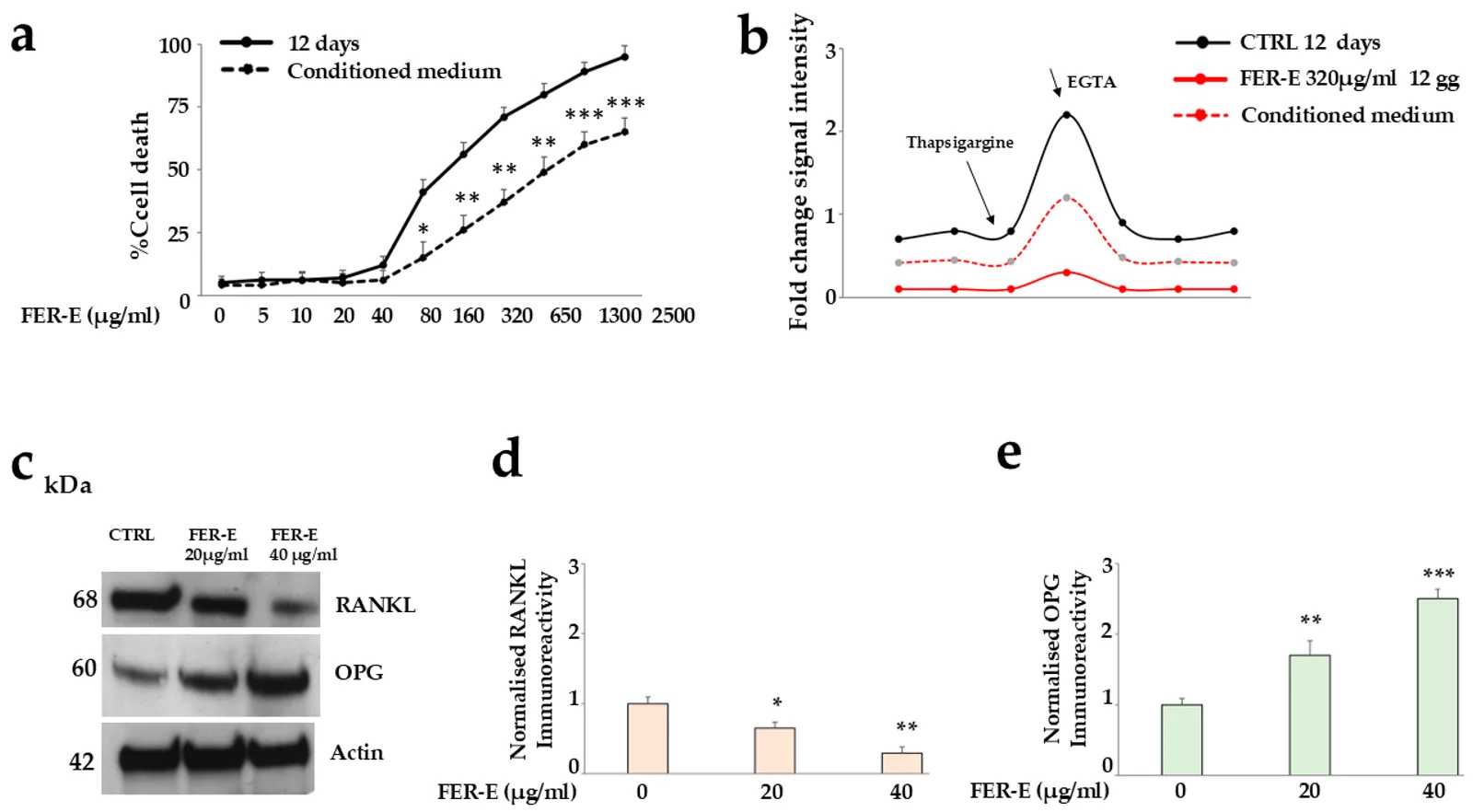
Effects of conditioned medium following treatment with FER-E for 12 days. Panel (**a**) shows the comparison of cell mortality of cells treated with FER-E for 12 days and cells treated equally, but subsequently also exposed to the conditioned medium for 24 h. Panel (**b**) highlights the concentration of cytosolic calcium in cells treated with FER-E for 12 days and cells treated equally, but subsequently also exposed to the conditioned medium for 24 h. Panel (**c**) shows the expression of RANKL and OGP proteins in cells treated with FER-E for 12 days and subsequently also exposed to the conditioned medium for 24 h. Finally, the respective quantifications are reported in panels (**d**,**e**). Three independent experiments were performed, and the values are expressed as the mean ± SD. * denotes *p* < 0.05 vs. untreated cells; ** denotes *p* < 0.01 vs. untreated cells; *** denotes *p* < 0.001 vs. untreated cells. A Tukey–Kramer comparison test followed the analysis of variance (ANOVA).

## Data Availability

Data are contained within the manuscript.
